# Aetiology of Vascular Purpura in a Single Centre Experience: Contribution of Clinical and Paraclinical Data

**DOI:** 10.31138/mjr.280723.aov

**Published:** 2024-01-29

**Authors:** Amira El Ouni, Faiza Ben Messaoud, Rym Khayati, C Abdelkafi, Zeineb Meddeb, Saloua Hamzaoui, Thara Larbi, Sana Toujani, Kamel Bouslama

**Affiliations:** CHU Mongi Slim, La Marsa, Tunisia

**Keywords:** vascular purpura, cutaneous leucocytoclastic vasculitis, aetiology

## Abstract

**Background:**

Vascular purpura can be the clinical expression of infectious, inflammatory, drug-related, neoplastic, and endocrine pathologies. To date, there is no consensus codifying the investigation of vascular purpura, especially when it is isolated.

**Patients and methods:**

We proposed to study through a retrospective study of 73 cases of vascular purpura, occurring during the period 2004–2019 in our internal medicine department, the contribution of various clinical and paraclinical data to the aetiological diagnosis of vascular purpura. Data were considered to be contributory only when they constituted a solid argument in favour of the aetiological diagnosis of vascular purpura.

**Results:**

Our series involved 73 patients including 41 women and 32 men (Gender ratio: 0.78). Mean age was 49 ± 17 years [16–80]. Vascular purpura was isolated in 3% of cases. For the remaining patients, it was associated with functional (91%) or physical (48%) manifestations. It was associated with other skin lesions in 45% of cases. The accepted aetiologies were primary vasculitis (26%), drug-related (15%), infectious (11%) and secondary to connectivitis (10%). No cause was found in a third of cases. Clinical data alone made it possible to suggest the aetiology in more than half of cases. Special investigations were contributory in 46% of cases. The course was contributory in 18% of patients for drug-related and paraneoplastic causes.

**Conclusion:**

vascular purpura’s diverse clinical presentation presents diagnostic challenges. Aetiologies include vasculitis, drug reactions, infections, and connective tissue disorders. Comprehensive clinical assessment is essential.

## INTRODUCTION

Purpura is a frequent reason for consultation in internal medicine. Although positive diagnosis is easy, the aetiological investigation can in some cases be laborious and costly. We are particularly interested in vascular purpura (VP), which may be the clinical expression of infectious, inflammatory, drug-induced, and neoplastic pathologies. However, it is possible that no aetiology is found. To date, there is no consensus guiding the investigation of VP. The objective of our work was to study the contribution of different clinical, biological, radiological, pathological, and evolutionary findings to the aetiological diagnosis of VP.

## METHODS

This is a retrospective, descriptive, monocentric study that included patients followed up for VP in our internal medicine department over the period going from January 2004 to August 2019 with platelet counts ≥ 50,000 elements/mm^3^^1^. We did not include in the study VPs that complicated infectious dermohypodermatitis, purpura simplex, Bateman purpura, Gardner-Diamond syndrome, or those purpuras due to hypercorticism, vascular hyperpressure, and purpuric capillaritis, because they do not require aetiologic investigation. Seven of our patients did not require an aetiological investigation of the purpura since it appeared after the aetiological diagnosis was made. These patients were not included in the contributing data section. We excluded from the study records that could not be used because the patients had been lost to follow up in less than three months. For each patient, we collected the clinical and paraclinical data at the time of the first attack of the purpuric lesions (for the patients who had a recurrence of the problem), the aetiological investigations performed, the treatment undertaken and the outcome. A finding was considered contributory only if its positivity constituted an argument in favour of the aetiological diagnosis of VP.

## RESULTS

### Study population

We identified 73 patients, 32 men and 41 women, giving a gender ratio of 0.78. Average age was 49 years with extremes ranging from 16 to 80 years. A peak of frequency was noted in the age group between 55 and 65 years.

**Figure 1.**
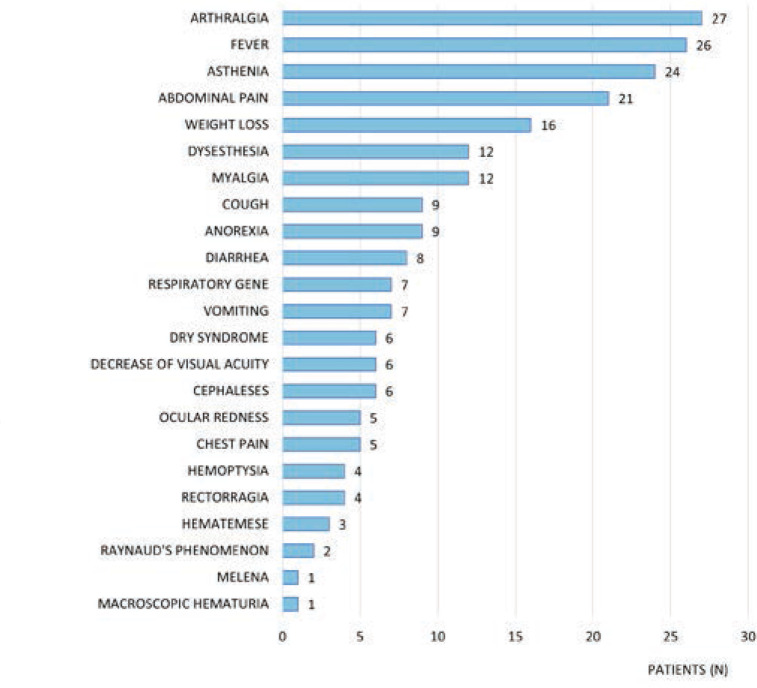
Functional signs associated with vascular purpura in our patients.

A personal history of manifestations preceding the onset of the first VP attack was reported by 65% of patients (n=43). The manifestations were cardiovascular (hypertension n=22; 33%, a stroke n=5; 8%, a valvular disease n=3; 5% and a cardiomyopathy n=3; 5%), or/and pleuropulmonary (asthma n=7; 11%). The histories also involved the ENT area: chronic rhinitis and/or nasal obstruction n=4; 6%, oral aphthosis, nasosinus polyposis, chronic sinusitis, and Widal syndrome one case each. Endocrinopathies (Type II DM); n=19; 29% and dyslipidaemia n=12; 18% were also reported as well as liver diseases (viral hepatitis, chronic alcoholic hepatitis, unspecified chronic liver disease) a case each (2%), and 2 cases (3%) of jaundice of unspecified aetiology. Two cases of epilepsy were also recorded as well as a history of chronic renal failure. Nineteen patients were smokers (29%). Eleven had hepatitis risk factors (17%) and two were chronic alcoholics (3%). The history of a viral contact with hepatitis C was found in one patient (active viral hepatitis C in the spouse). A possible triggering factor was found in 44% of patients. In 29% of the cases, it was a drug and/or herbal treatment that preceded the onset of VP and the iatrogenic origin was accepted in 58% of the cases. For two patients, the drug origin was definitive and for nine others, it was probable. Antibiotics were the most common cause (55%), particularly beta-lactam antibiotics. An episode of ENT infection preceded the onset of VP in ten patients (15%). On the first visit, the VP was isolated in three patients (5%). In other patients, it was associated with other functional signs as detailed in **Figure 1**. The purpura developed acutely in 58 patients (80%) and chronically in the remaining 15 others (20%).

Clinically, the VP was infiltrated in 64% of cases. Necrotic and bullous appearances were noted in 41 and 15% of patients respectively. The purpura was pruritic in 21% of cases. Lesions predominated in the lower limbs in 93% of patients and were consistently bilateral in this location with extension to the feet (6%), legs (35%), thighs (46%), and buttocks (13%). The purpura was diffuse (≥2 locations) in 68% of cases. In addition, other skin manifestations were found in 33 patients (45%), seven of whom had more than two associated lesions. These were: nonspecific erythematous lesions (n=10), livedo (n=8), skin ulcerations (n=7), pustules (n=5), subcutaneous nodules (n=4), oral ulcerations, digital necrosis and fixed urticaria in two cases each. Physical signs were found in 32 patients (48%) as summarised in **Table 1**. Ophthalmoscopic and ENT examinations were performed in eleven and six patients respectively and were found to be pathological in seven and three patients, respectively.

**Table 1.** Physical signs associated with VP in our patients.

**PHYSICAL SIGNS**	**PATIENTS (%)**

**Respiratory signs**	**14 (21%)**
**-Polypnoea**	8 (12%)
**-Auscultatory rales**	4 (6%)
**-Pleural syndrome**	2 (3%)

**Osteoarticular signs**	**12 (18%)**
**-Arthritis**	

**Neurological signs**	**12 (18%)**
**-Altered consciousness**	7 (11%)
**-Localisation signs**	8 (12%)
**-Meningeal syndrome**	1 (2%)

**Cardiovascular signs**	**10 (15%)**
**-Altered hemodynamic status**	2 (3%)
**-Heart murmur**	6 (9%)
**-Other auscultatory abnormalities**	2 (3%)

**Abdominal signs**	**9 (14%)**
**-Abdominal tenderness**	9 (14%)
**-Pain on lumbar shaking**	3 (4%)
**-Intra-abdominal mass**	2 (3%)

**Other**	
**-Superficial adenopathies**	13 (20%)
**-Oedema of the lower limbs**	8 (12%)
**-Hepatomegaly**	5 (8%)
**-Conjunctival jaundice**	4 (6%)
**-Oedema of the upper limbs**	3 (5%)
**-Splenomegaly**	3 (5%)
**-Thyroid nodules**	2 (3%)
**-Gingival hypertrophy**	1 (2%)
**-Exophthalmos**	1 (2%)
**-Collateral venous circulation**	1 (2%)
-Proteinuria and/or microscopic haematuria ^*^	**22 (36%)**

* The search for significant haematuria and/or proteinuria by urine dipstick was performed in 92% of patients.

Electrocardiogram (ECG) was performed routinely in 64 patients (97%) and revealed electrical abnormalities in 24 patients (38%): repolarization disorders (n=17), conduction disorders (n=7) and rhythm disorders (n=6). Wolff-Parkinson-White syndrome was discovered incidentally. EMG was performed in seven patients with a peripheral neurogenic syndrome. It showed a sensory-motor axonal neuropathy in four cases (57%). **Table 2** summarises the frequency of the various laboratory tests performed and the frequency of the pathological findings. Tumour markers were ordered for seven patients (11%). They included prostate-specific antigen (n=4), alpha-fetoprotein (n=2) and CA-125 (n=1). Midstream urine specimen (MSU), ova and parasites study in stool, blood cultures and cerebrospinal fluid (CSF) were performed in 48 (73%), four (6%), ten (15%) and three (4%) patients respectively and were pathological in 19%, 25%, 30% and 67% respectively. A total of 100 serologic tests were requested for all patients mainly, those for hepatitis B virus (n=30), hepatitis C virus (n=28), HIV (n=9), parvovirus B19 (n=7), and cytomegalovirus (n=4). Only HCV and CMV tests were positive in 11% and 25% of cases, respectively. Other serologic tests were performed for brucellosis (n=3), syphilis (n=3), Epstein-Barr virus (n=2), rickettsioses (n=2), toxoplasmosis (n=2) and in one case each for rubella, coxsackies, Lyme disease, leishmaniasis, toxocariasis, and hydatidosis. Skin biopsies were ordered for 26 patients (39%). The median time to completion of the skin biopsy in relation to the onset of the VP was 12 days (10–24 days) with extremes ranging from 3 to 90 days. Histologic abnormalities were observed in all the patients who had had a VP biopsy. Leukocytoclastic cutaneous vasculitis was observed in 24 cases (92%). The infiltrate was predominantly neutrophilic (61%), neutrophilic and eosinophilic (19%), lymphocytic (19%) or predominantly eosinophilic (1%). In two patients, there were purpuric lesions without vasculitis (extravasation of red blood cells, thickening and oedema of the vascular walls without leukocytoclasia). The other biopsies performed during the aetiologic investigation involved the kidneys (n=7) which proved to be pathologic in all cases, the GI system (n=4) which was pathologic in 50% of cases, the accessory salivary glands (n=3) that were pathologic in 67% of cases and the lymph nodes (n=2) that were also pathologic in 50% of cases. Recto-colon and bone marrow biopsies (n=2) revealed no abnormalities. Cavum, breast, nasal mucosa, temporal artery, and liver biopsies were always pathological. Direct cutaneous immunofluorescence (DIF) was studied in 21 cases (81%) with the presence of deposits on the vascular walls or at the dermal-epidermal junction in 11 cases (52%). These were immunoglobulin deposits (IgA 48%; IgM 19% IgG 14%) and C3 (48%) and C1q (9%) complements. DIF systematically coupled with renal biopsy was performed with a frequency of 11% (n=7) revealing extra-capillary glomerulonephritis in one case and mesangial proliferation with IgA (67%) and C3 (33%) deposits in six cases. As for the immunological tests, their results are summarised in **Table 3**.

**Table 2.** Frequency of performance and pathological results of laboratory tests in patients with VP in our study.

**Laboratory tests**	**Frequency of realisation in %**	**Frequency of pathological findings in %**
**CBC**	99	57
**ESR**	83	62
**CRP**	79	52
**Procalcitonin**	3	50
**Creatinine**	97	11
**Serum electrolytes**	94	15
**24-hour proteinuria**	44	41
**PEP**	70	61
**Triglyceridaemia**	73	21
**Cholesterolaemia**	73	33
**γGT/ALP**	70	37
**Transaminases**	70	22
**LDH**	68	47
**CPK**	61	10
**Serum calcium levels**	41	7

CBC: complete Blood count; CRP: C-reactive protein; ESR: erythrocyte sedimentation rate; PEP: protein electrophoresis; PT/ATT: prothrombin time/activated thromboplastin time; γGT/PAL: gamma-glutamyl transferase/alkaline phosphatase; LDH: lactate dehydrogenase; CPK: creatine phosphokinase.

**Table 3.** Frequency of performance and pathological results of immunological tests in patients with VP.

**Immunological tests**	**Frequency of performance**	**Positivity rate**
ANA	67%	27%
Anti-DNA native antibodies	30%	20%
Anti-ENA antibodies	29%	11%
Anti-CCP antibodies	8%	20%
Cryoglobulinemia	49%	6%
ANCA	68%	9%
Rheumatoid factor	9%	50%
Complement C3/C4	61%	15%
Serum IgA	12%	50%
Anti-phospholipid antibodies^*^	14%	33%

ANA: antinuclear antibodies; ENA: extractable nuclear antigen; CCP: cyclic citrullinated peptides; ANCA: Anti-Neutrophilic Cytoplasmic Autoantibody

* Lupus anticoagulant, anti-cardiolipin antibodies and anti-β2-glycoprotein antibodies I

The aetiologic distribution of VP in our patients is detailed in **Table 4**. The frequency of secondary vasculitides was 38%, mainly of drug origin. Primary vasculitides represented 26% of the aetiologies.

**Table 4.** Aetiologies of VP in our patients.

**AETIOLOGIES**	**PATIENTS (%) n=73**

**Purpura with vasculitis**	

**PRIMARY VASCULITIDES**	**19 (26%)**

*IgA vasculitis*	15
*Eosinophilic granulomatosis with polyangiitis*	2
*Granulomatosis with polyangiitis*	2

**SECONDARY VASCULITIDES**	**28 (38%)**

**Drug induced**	**11 (15%)**
**Infectious**	**8 (11%)**
*Infectious endocarditis*	3
*Viral hepatitis C associated with cryoglobulinemia*	3
*Meningococcaemia*	1
*Cytomegalovirus infection*	1
**Secondary to connectivitis**	**7 (10%)**
*Systemic lupus erythematosus associated with Sjögren’s syndrome*	3
*Neurolupus*	1
*Waldenström’s hypergammaglobulinaemic purpura (Sjögren’s syndrome)*	2
*Dermatomyositis*	1
**Sarcoidosis vasculitis**	**1 (1%)**
**Neoplastic**	**1 (1%)**
Renal tumour	1

**Purpura without vasculitis**	

*Antiphospholipid syndrome*	**1 (1%)**
*Sneddon’s syndrome*	**1 (1%)**
*AL amyloidosis*	**1 (1%)**

**TOTAL**	**50**

**UNDETERMINED CAUSES**	**23**

**Figure 2.**
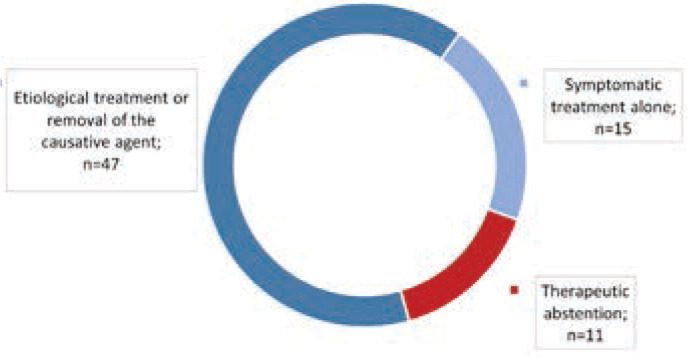
Treatments prescribed for patients with vascular purpura.

**Figure 2** summarises the different treatments prescribed for all our patients.

The course was marked by regression of the VP in 56% of cases (20% spontaneously and 36% on aetiologic treatment), followed by a recurrence in 17% of cases (the median time to recurrence was 7 months with extremes ranging from 2 to 97 months). Fifteen per cent of the patients were lost to follow-up with a median time to follow-up of 12 months. Four per cent of the VPs persisted, and in 8% of the cases, death occurred as a result of extension of the lesions.

Regarding the degree of contribution of the findings provided by physical examination and by special investigations, family history was not useful. Personal history contributed to the diagnosis in 9% of patients. These were eosinophilic granulomatosis with polyangiitis (EGPA) with uncontrolled asthma, chronic rhino-sinusitis, and/or naso-sinusal polyposis (n=2) and infective endocarditis suggested by the history of aortic disease and valvular disease (n=2). Lifestyle data were only helpful in one patient, namely, the history of viral contamination by hepatitis C (active hepatitis C in the spouse) directing the diagnosis to a cryoglobulinemia secondary to HCV. The history of the disease was contributory to the diagnosis in 47% of patients. The history of drugs and/or herbs intake before the onset of the VP was contributory in 17% of patients, all of whom had purpuras due to drug-induced vasculitis. The history of an ENT infection preceding the VP guided the diagnosis in 11% of patients. All cases were of IgA vasculitis. Symptoms contributed to the diagnosis in 20 patients (30%). Indeed, IgA vasculitis (n=12) was suggested by abdominal pain, gastro-intestinal manifestations (vomiting, diarrhoea, digestive haemorrhage) and/or macroscopic haematuria. EGPA (n=2) was suggested by neurological (dysesthesia) and respiratory signs (respiratory discomfort, haemoptysis), progressive deafness and/or chest pain. Neurolupus (n=1) was suspected in the presence of neurological manifestations (headache, dysesthesia, vertigo of central origin). A Sjögren syndrome (SS) was considered in front of ocular and oral dryness. Septic causes (n=4) (meningococcaemia and infective endocarditis) were suspected when fever and chills were present. Skin examination was contributory in 6% of patients. Purpura fulminans was suspected due to the typical necrotic appearance and the rapid expansion of the purpura. Skin lesions associated with VP suggested the diagnosis in three patients. EGPA was suspected in the presence of multiple subcutaneous nodules, infective endocarditis in the presence of digital necroses, and anti-phospholipid syndrome in the presence of livedo and digital necroses associated with purpura. Physical signs associated with VP were contributory in 26% of patients. The diagnosis was directed towards EGPA in two patients by neurologic, respiratory and/or cardiac signs. IgA vasculitis (n=8) was suggested by the presence of arthritis, haematuria, and/or proteinuria on urine dipstick. Infective endocarditis (n=3) was suggested by sepsis, heart murmur and/or splenomegaly. Meningococcaemia was revealed by a meningeal syndrome. Cryoglobulinaemia secondary to HCV was suspected in front of hepatomegaly and a conjunctival icterus, a neurolupus, among others, in front of neurological localisation signs and CMV infection in front of hepatomegaly. Special investigations were contributory in 18% of cases. The ophthalmoscopic examination showed a dry syndrome suggesting SS in two cases and the ENT examination showed a nasosinusal polyposis and a bilateral sensorineural hearing loss, suggesting EGPA. The remaining patients had abnormalities unrelated to the aetiology of VP.

ECG was contributory in only one case, showing electrical signs of myocarditis in a patient with EGPA. The EMG was contributory in 43% of cases. It showed a sensory-motor axonal neuropathy in four cases (57%), pointing in two cases to EGPA and in one case to granulomatosis with polyangiitis. In one patient, the EMG was pathological without however suggesting the diagnosis. This was a systemic involvement in the course of a drug-induced vasculitis. The contribution of biochemical and haematological examinations to the aetiological investigation was estimated at 4%. Only CBC, creatinine, calcium, 24-hour proteinuria, and protein electrophoresis were contributory to the aetiologic diagnosis in two, five, four, thirty-four, and four percent of cases, respectively. The CBC was contributory by the presence of a major hypereosinophilia pointing to EGPA (n=2) and to a drug-induced origin (n=1). Positive 24-hour proteinuria in 15 patients and elevated creatinine in five cases were indicative of IgA vasculitis. Hypergammaglobulinemia with a polyclonal appearance on protein electrophoresis pointed to Waldenström’s hypergammaglobulinemic purpura and sarcoidosis. Hypercalcemia referred to sarcoidosis in one case. Tumour markers did not contribute to the aetiological diagnosis in any patient. Bacteriological examinations contributed in 3% of cases to the aetiological investigation of VP. Blood cultures, whose contribution was estimated at 10%, pointed in one case to a coagulase-negative Staphylococcus infective endocarditis. In two other patients, they had no orientation value (Escherichia coli bacteraemia concomitant with EGPA and positive blood cultures for yeasts in a patient with IgA vasculitis). The CSF study contributed to the aetiological diagnosis in 33% of cases. The diagnosis of meningococcaemia was confirmed by a CSF study (Neisseria meningitidis +) in one patient. MSU and ova and parasites study in stool examinations were not contributory. The contribution of the serologic tests to the aetiological diagnosis was estimated at 4%. HCV and CMV tests were contributory whenever they were positive. The serologic tests for ASLO/ASDOR serology were not contributory. The contribution of anatomicopathological examinations (excluding IFD) was estimated at 16%. Skin biopsy was contributory in only one patient (leukocytoclastic vasculitis with a majority eosinophilic infiltrate pointing to EGPA). Accessory salivary glands biopsy pointed to SS in two patients (contribution estimated at 64%). Liver biopsy was contributory in one patient showing tuberculoid granulomatosis without caseous necrosis pointing to sarcoidosis. Skin DIF was contributory in 38% of cases (n=8 IgA deposits pointing to IgA vasculitis). IFD systematically paired with renal biopsy was contributory in 83% of cases. These were glomerulonephritis with mesangial IgA (67%) and C3 (33%) deposits in relation to IgA vasculitis (n=6) and in another case it was pauci-immune extra-capillary glomerulonephritis in relation to granulomatosis with polyangiitis. Immunological tests contributed to the aetiological diagnosis in 18% of cases. Antinuclear antibodies (ANA), anti-native DNA antibodies and anti-ENA antibodies were contributory in 5% of cases each. Anti-CCP antibodies were not contributory. The test for cryoglobulinaemia was contributory in 6% of cases. The search for ANCA, rheumatoid factor, anti-phospholipid antibodies, complement C3/C4 and serum IgA were contributory in 7%, 17%, 11%, 3% and 25% of cases, respectively. The course was contributory to the aetiological diagnosis in 18% of patients. The purpuras were drug-related in 11 cases and of paraneo-plastic origin in one patient.

## DISCUSSION

We noticed that despite the many special investigations ordered, the main diagnostic contribution remains that of clinical data (history-taking and physical examination) hence the importance of a well conducted history and of a meticulous examination in order to direct further investigations. As the aetiological assessment is not consensual, our work deserves credit for being interested in the contribution of the various clinical, biological, radiological and pathological elements in order to facilitate the aetiological approach in front of a case of VP, especially if it is isolated. However, the retrospective character of the study remains a limiting factor because of the numerous missing data in relation to patients lost to follow-up. Moreover, a selection bias in favour of inflammatory diseases compared to infectious and neoplastic causes should be considered.

Few studies in the international literature have focused on the entity of VP alone. The majority of studies have focused on all cutaneous leukocytoclastic vasculitides, the most frequent clinical manifestations of which is VP (47 to 98% of cases depending on the series).^2–12^ In Tunisian series, the frequency of VP is between 2.5 and 9.36 new cases/year with an average of five cases per year.^13–16^ In our series, we identified 73 patients with VP over 16 years, i.e., an average of five new cases per year. The frequency of new cases in two Indian and French series was respectively 18 and 87 new cases/year. The gender ratio varies in the literature between 0.93 and 1.34.^3–18^ A slight female predominance was noted in our series (gender ratio = 0.78). The mean age ranged from 34 to 60 years with extremes of one to 95 years.^4–18^ Our data agree with those of the literature (mean age = 49 years) with a peak of frequency concerning the 55–65 years age group.

The contribution of history-taking was greater than that of physical examination. Data on the history of the disease were the most contributory. The history of drug-intake before the onset of VP varies with a rate of 5 to 37% in the literature.^3,5,6,9,11^ In our series, this rate was 26%. Betalactam antibiotics were the most commonly incriminated drugs in our study. This notion has been found in some series.^1,19,20^ In other studies, a predominance of non-steroidal anti-inflammatory drugs has been noted.^3,7,8^ An episode of ENT infection preceding the appearance of skin lesions is frequently reported in the literature with a rate varying from 14 to 34%.^4,5,9,11^ In our series, this rate was 14%. Symptoms and signs are indicative of the disease in almost all of our patients (95%). The VP was rarely isolated. Referral signs may appear secondarily, hence the importance of a history and of periodic somatic examinations. The acute mode of onset and course is the most frequent in the Tunisian literature.^5,14–16^ This result is verified in our series.

Purpura was often infiltrated in our series as well as in the literature. This is explained by a predominance of purpura due to vasculitis. The frequency of necrotic, bullous, and pruritic aspects varies from one series to another.^5,14,16^ The semiological features of purpura were contributory in one patient in our series, pointing to purpura fulminans. The lower limbs are the preferential site for VP in all series in the literature with rates ranging from 44 to 90% versus 93% in our series. VP is diffuse with a frequency ranging from 20 to 52% according to the literature vs 68% in our series.^3,5,13–16,18^ We suspected a selection bias given the predominance of inflammatory diseases in our series. However, the link between the diffuse nature and the systemic involvement is controversial.^21,22^ Large-scale studies would allow confirmation or refutation of this correlation. The type of skin lesions associated with VP is closely related to the size of the vessels affected by the vasculitis. This is illustrated by **Table 5**. This medical fact allows us to suspect certain aetiologies.^21^ Indeed, necrotising vasculitides of medium calibre arterioles are suggestive of periarteritis nodosa without nonetheless being specific.^23^ Other cutaneous manifestations would be significantly associated with certain types of ANCA-associated vasculitides, notably urticaria and subcutaneous nodules during EGPA and ulcers during granulomatosis with polyangiitis.^24^ These VP-associated skin lesions suggested the diagnosis in 5% of our patients. As for the physical signs associated with VP, arthritis would be the most frequent (16–45%)^13–16^ vs 18% in our series. Urine test strip abnormalities are also frequently noted with rates ranging between 29 and 45%^3,12,13,16^ vs 36% in our series. According to Ioannidou et al., up to half of patients with leukocytoclastic vasculitis may have renal involvement.^25^ Francès et al. recommend that urinary examinations be repeated at a rate of once a week to once a month for a minimum of three months or even for a whole year.^23^ The ENT region is a preferential site for biopsy in cases of suspected granulomatosis with polyangiitis if the examination reveals granulomas. Ophthalmologic involvement may be of great value in case of a specific lesion.

**Table 5.** Cutaneous vasculitis and size of vessels affected.

**Morphologic examination**	**Contribution**

**ENDOSCOPIES**	**27%**
**oeso-gastroduodenal Fibroscopy**	13%
**Bronchoscopy**	0%

**Colonoscopy**	25%

**Cutaneous vasculitis**	**Fixed urticaria**	**Non-specific erythematous lesion**	**Vesicle**	**Pustule**	**Livedo**	**Skin ulcer**	**Subcutaneous nodule**	**Digital necrosis**
**Damage to small-calibre vessels**	☒	☒	☒	☒	☒			
**Damage to medium-calibre vessels**					☒	☒	☒	☒

Although it has had little value in directing the aetiology of a VP, ECG remains an essential examination procedure in routine practice. It can reveal serious electrical disturbances and prevent serious complications. EMG has shown a peripheral neuropathy in 5.6–38.16% of cases during the aetiological assessment of VP^5,16^ against 6% in our series. It would be of no interest in the absence of a clinical context. The almost systematically requested laboratory tests contributed in 4% of cases to the aetiologic investigation. But let us not forget that we have chosen to study the contribution of positive data. Indeed, a biological inflammatory syndrome during VP has no aetiologic value. Its absence, on the other hand, points to a drug-related origin. Major blood hypereosinophilia points to EGPA.^26^ It is also frequent in systemic drug-induced vasculitis.^23^ Tumour marker assays (11%) were all negative. We were unable to compare our findings with the literature results due to a lack of similar data. In most cases, they are only useful for directing the follow-up treatment and for predicting the outcome. Their importance is therefore limited during the aetiological assessment of a VP. Urine culture, a laboratory test, very frequently requested in hospitalised patients, was in no way contributory. Its routine prescription has no diagnostic or therapeutic value in this context. Current recommendations do not recommend the treatment of asymptomatic bacteriuria, except in exceptional cases.^27,28^ The respective contribution of hepatitis B and C serum tests were nil and 11%. Positive viral C serum test was consistently associated with cryoglobulinemia. It could be concluded, subject to broader spectrum studies, that requesting hepatitis C serum test is more useful in cases of positive cryoglobulinemia or in association with suggestive clinical features. It should also be noted that despite a 14% frequency of HIV serum test, no case of HIV secondary to VP was found. This result calls into question the usefulness of systematically requesting serologic tests for HIV in this context. Chest X-ray, which was performed almost systematically, had a good diagnostic value when it showed abnormalities (frequency of abnormalities 34% vs contribution 20%). Other less accessible imaging investigations were most often based on clinical findings. The usefulness of skin biopsy without immunofluorescence remains disputed, particularly in the presence of infiltrated purpura.^22,23^ In our case, it was contributory in only one patient (contribution 4% vs 38%). The study by Lath et al. evaluating the diagnostic utility of IFD in the face of cutaneous vasculitis confirms a considerable contribution to the diagnosis of IgA vasculitis but also to lupus vasculitis.^30^ Thus, any suspicion of cutaneous vasculitis should indicate DIF. For other biopsies, the more accessible and less invasive salivary gland biopsy, performed in targeted patients, would be useful for the diagnosis of SS and amyloidosis. It is of moderate interest for sarcoidosis and absent for other autoimmune diseases.^29^ The contribution of renal DIF was 83% in our series because it was systematically oriented. It should be noted that it may be contributory by its negativity in pauci-immune vasculitides. For immunological tests, the systematic search for cryoglobulinemia and ANCA-associated vasculitis may be justified. The search for ANA, native anti-DNA, anti-ENA, and hypocomplementemia, which are not very helpful, should be done in a suggestive context.

**Table 6** summarises the causes of cutaneous vasculitis according to the literature reviews.

**Table 6.** Causes of cutaneous vasculitis according to literature reviews.

**Series**	**Primary vasculitis**	**Connectivitis**	**Infectious vasculitis**	**Drug-induced vasculitis**	**Neoplastic vasculitis**	**Undetermined**	**Ref**
**Carlson et al. (Meta-analysis)** n=2161	NS	11.70%	22,50%	20,10%	4,30%	39,00%	[40]
**Aounallah et al.** n=85	25%	6%	13%	6%	4%	35%	[5]
**Al-Mutairi et al.** n=57	18%	5%	14%	18%	2%	37%	[6]
**Klii et al.** n=48	51%	8%	2%	8%	0%	31%	[13]
**Frikha et al.** n=66	29%	12%	18%	9%	0%	29%	[14]
**Ben Yahia et al.** n=44	52%	7%	4%	2%	2%	27%	[15]
**Laanani et al.** n=131	32,0%	23,0%	13,0%	3,8%	0,8%	20,6%	[16]
**Toujani et al.** n=58	38%	10%	7%	7%	7%	26%	[18]
**Hachi A.** n=55	NS	NS	18%	9%	NS	27,3%	[32]
**Samson et al.** n=112	28,6%	19,6%	6,3%	4,5%	7,1%	33,9%	[10]
**Arora et al.** n=84	39%	4%	10%	1%	0%	35%	[4]
**Tai et al. n=93**	23%	9%	22%	17%	2%	22%	[2]
**Swapna et al.** n=66	12%	3%	18%	23%	2%	39%	[8]
**Our series**	**26%**	**10%**	**11%**	**15%**	**1%**	**32%**	-

NS: Not specified.

* We have recalculated the figures according to our aetiological classification. These calculations remain approximate.

The frequency of purpuras in the context of primary vasculitis varies between 32 and 52% vs 26% in our series. ^15,16^ Indeed, the Tunisian studies were mainly carried out in internal medicine departments; elsewhere, several studies were conducted in dermatology or endocrinology departments. IgA vasculitis is the most common cause of VP among primary vasculitides in the literature.^4–18,39^ A meta-analysis published in 2005 in the United States studying the frequency of different aetiologies of cutaneous vasculitides (n=2161) revealed a mean rate of IgA vasculitides of 10%^40^ vs 21% in our series, a selection bias. The frequency of VP during ANCA-associated vasculitis varies between nil and 19% according to the series vs. 5% in our study.^4–16,18,39–41^ According to a cross-sectional study (n=1184), vascular purpura is the most frequent skin involvement in ANCA-associated vasculitis and often appears early.^42^ Its presence would be an indicator of the activity of the vasculitis.^31^

Concerning VP of secondary origin, a drug-related cause is found in 1 to 23% of cases, compared with 15% in our study.^2,4–11,13–16,18,32^ This variability is due to the criteria used to determine the responsibility of the incriminated drug.^1^ Cutaneous vasculitis of infectious origin is the most common cause of death in the meta-analysis by Carlson et al. with a mean rate of 12%.^40^ This rate was of 11% in our series. It is only rarely demonstrated. We have included in our study only infections with a clearly established role in the genesis of secondary vasculitis; the most documented being bacteraemia and infections with HCV, HBV, HIV, CMV, and parvovirus B19.^42^ Purpuric eruptions associated with SARS-CoV-2 have been described in recent studies, with the primary mechanism being hypersensitivity to the virus.^43^ VP is the first manifestation of meningococcaemia.^44^ Only one case was reported in our series. This result is consistent with the average rate of 1.2% of cutaneous vasculitis secondary to severe sepsis in the meta-analysis by Carlson et al.^34^ We noted three cases of VP heralding infective endocarditis (4%) vs 0.8% in the series by Loricera et al.^12^ VP secondary to CMV infection is rarely reported in the literature. We had one case in our study. Although the thrombocytopenic origin of purpura in this infection is commonly accepted,^45^ recent data have demonstrated its vasculitic origin.^46,47^ It should be noted that cutaneous manifestations during CMV infections are mainly exclusively seen in immunocompromised subjects,^42^ but our patient was immunocompetent. In the meta-analysis by Carlson et al.,^40^ the mean rate of cutaneous vasculitis secondary to viral hepatitis was 3.1% vs. 4% in our series. According to methodologically rigorous studies, 55–95% of the patients with symptomatic mixed cryoglobulinemia have anti-HCV antibodies in their serum. In addition, the prospective cohort follow-up of HCV-affected patients has shown the presence of mixed cryoglobulinemia in 36–55% of cases.^48^ However, the biological abnormality (cryoglobulin positivity) should not be confused with the clinical manifestations of cryoglobulinaemic vasculitis. We did not find any cases of VP secondary to viral hepatitis B in our study. Although much rarer than during HCV infection, mixed cryoglobulinaemias during hepatitis B have been described, accounting for less than 10% of the causes of mixed cryoglobulinaemias apart from HCV infection.^49^ We did not identify any cases of VP secondary to HIV in our series, which is reportedly rare.^40^ Vasculitides secondary to HIV are thought to have a neurological rather than a cutaneous tropism and to occur at an advanced stage of the disease.^23^

The frequency of VP secondary to connective tissue diseases varies in the literature between 6% and 23% vs 10% in our study.^7,13–16,39,50^ VP associated with systemic lupus erythematosus is reported with rates ranging from two to nine percent in the literature.^13–16,18^ Our results were comparable (5%). We found two cases of Waldenström hypergammaglobulinaemic purpuras associated with SS (3%). This aetiology is rarely reported in the literature. Actually, it is a diagnosis of exclusion that requires an exhaustive aetiological work-up. Its association with a connective tissue disease is frequently reported.^51^ We noted one case of VP in the context of dermatomyositis in our study. VP is rarely reported in the literature in this context. Cutaneous vasculitis during dermatomyositis would be suggestive of an associated neoplasia.^52^ VP associated with sarcoidosis, although rare and non-specific, has been described in the literature.^53–55^ We have counted one case. VP in the context of neoplasia is very rare in the literature.^2,3–16,18,40^ Only one neoplastic cause was documented in our series (renal cancer). The skin lesions had completely disappeared after lumpectomy. Concerning purpuras without vasculitis, isolated cases of VP in the context of anti-phospholipid syndrome have been described in the Tunisian literature ^5,18^. Their mechanism is thrombotic. We noted only one case in our study. A case of Sneddon syndrome associated with VP was published in 2018 in the Journal Rheumatology Advances in Practice in the United Kingdom. In this published case, the patient additionally had an IgA nephropathy.^56^ A case of VP in the context of AL amyloidosis was found in our study. Periorbital purpura is a pathognomonic but late sign occurring in 10 to 15% of cases.^57,58^ Amyloid purpura can also be seen in a supramammary location,^59^ as was the case in our patient. It is rarely indicative of AL amyloidosis.^60^ In our case, the diagnosis had already been made before its appearance. However, two studies from China and Portugal describe the first appearance of purpura, the biopsy of which led to the diagnosis of AL amyloidosis before the appearance of other systemic disorders.^61,62^

Despite extensive investigations, the proportion of undetermined causes of VP in the literature is 10 to 45%.^5,13,16,18^ It was 32% in our series.

The course was contributory in 18% of patients for purpuras of drug and paraneoplastic causes. Regression of the lesions is the rule.^13,14,16^ Lesion regression without recurrence is often the rule after treatment of the underlying aetiology.^63^ A lesion recurrence may, in some cases, reflect a relapse of the underlying disease. Recurrences are frequent in the literature (18–40%). They represented 17% of cases in our series. The average time to recurrence varies from 3 to 14 months in some series.^10,16^ It was seven months in our series. The persistence of skin lesions over time may be related to insufficient follow-up time which varied according to the series reported from two to six years.^4,10,16^ It was 12 months in our series.

### Treatment

Symptomatic complaints such as pruritus or burning in purpura vasculitis can be managed through a combination of strategies, including bed rest, warming, and elevating the lower extremities. Additionally, nonsteroidal anti-inflammatory drugs (NSAIDs), analgesics, and antihistamines are employed^64^ as well as corticosteroids in severe cases. In some instances, medications to suppress the immune system may be considered. It’s important to note that certain medications within this group have been associated with causing vasculitis, and if suspected in specific cases, they should be avoided.^64^ The specific treatment approach depends on the severity of the condition and its underlying causes. Close medical monitoring is often necessary to ensure the best outcome for individuals with VP.

## CONCLUSION

Our work has shown that VP is rarely isolated and clinical data are highly contributory to the aetiological investigation. Actually, in cases where history-taking and physical examination do not directly lead to the diagnosis, they can be used to suggest it and to guide the investigations. When VP is isolated, direct immunofluorescence on skin biopsy, IgA assay, cryoglobulinaemiam and hepatitis B and C serum tests in case of positivity of cryoglobulinaemia, are the most contributory tests. These data need to be confirmed by larger prospective studies.

## Figures and Tables

**Figure 3.**
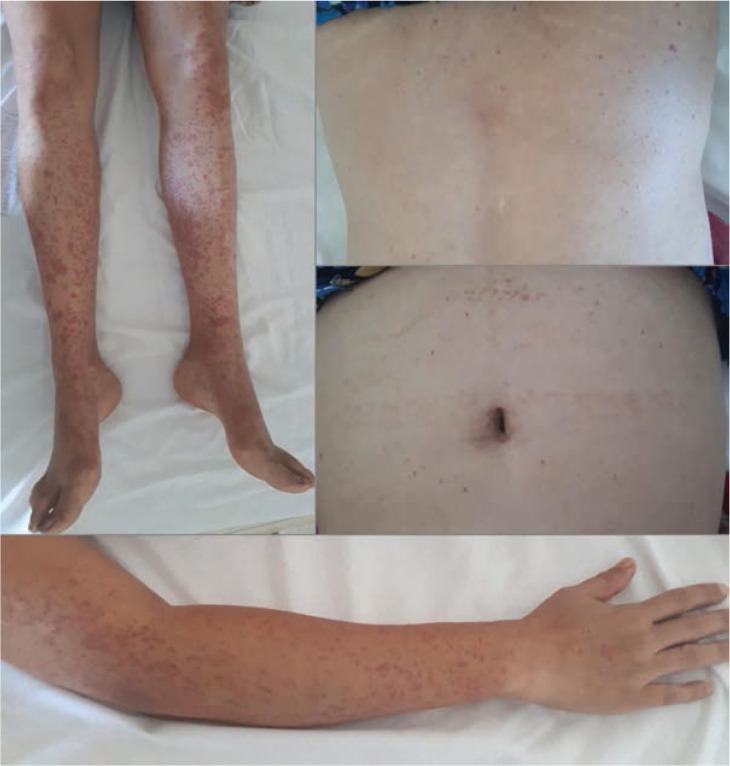
Diffuse vascular purpura, petechial, confluent in places, of undetermined etiology.

**Figure 4.**
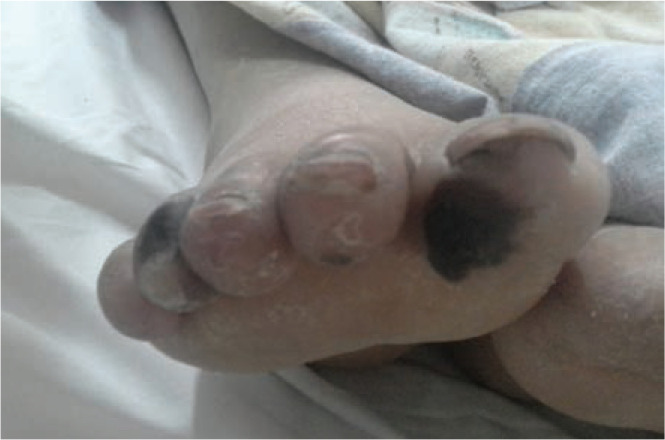
Necrotic purpura of the right forefoot in Sneddon’s syndrome.

**Figure 5.**
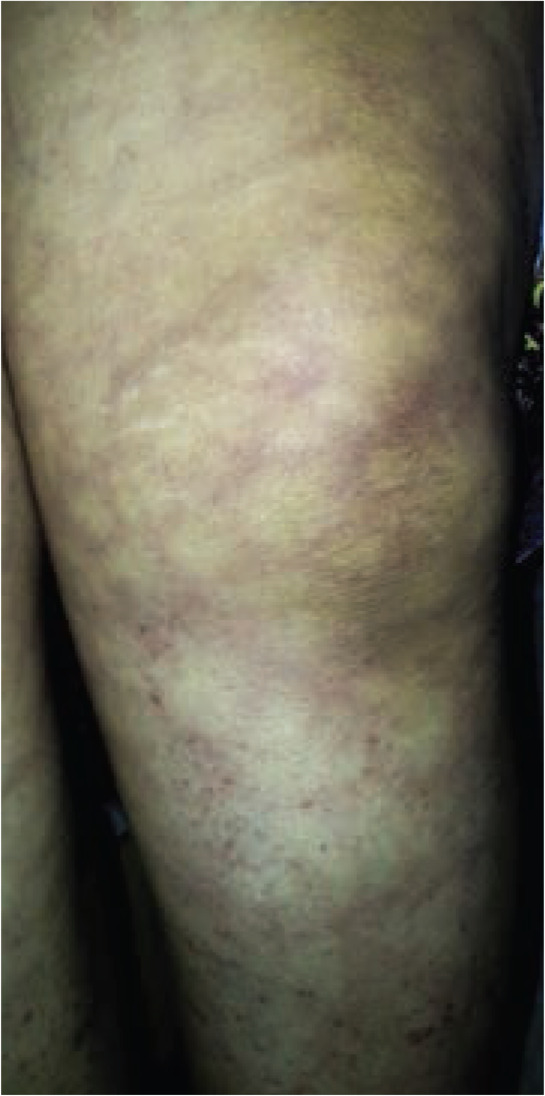
Livedo reticularis associated with purpura, on the knees, in the context of drug-induced vasculitis.

**Figure 6.**
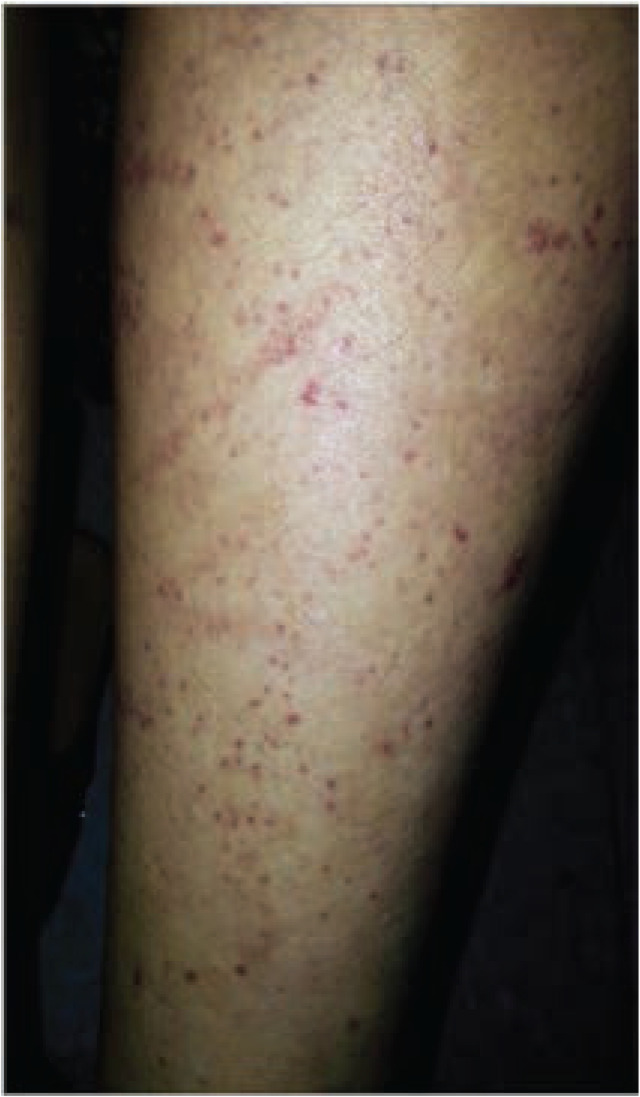
Petechial and linear vascular purpura of the lower limb caused by drugs.

**Figure 7.**
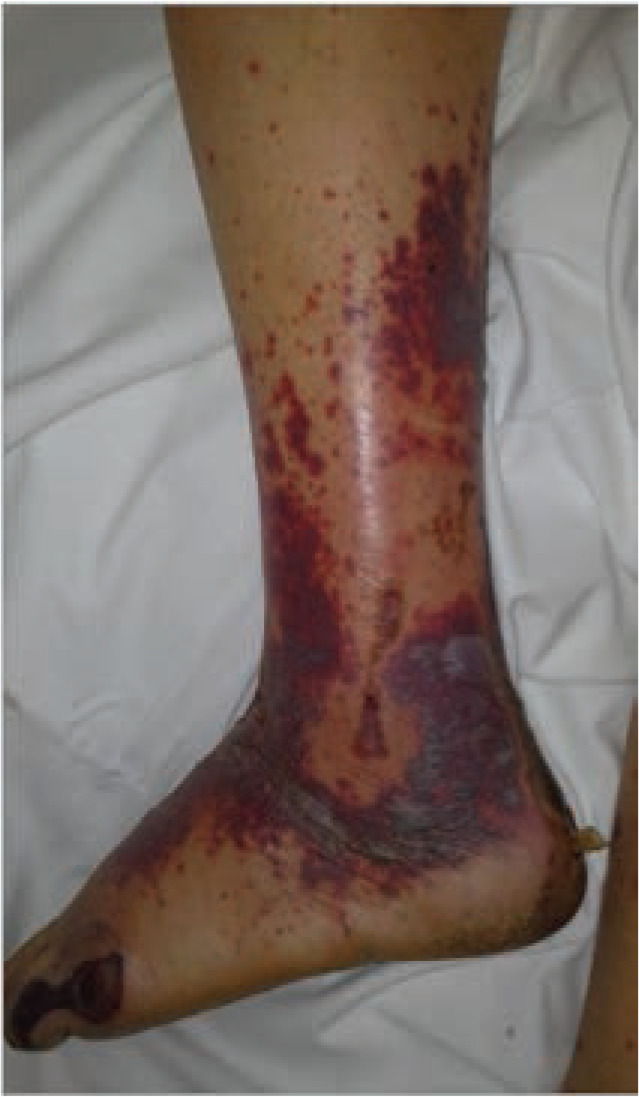
Polymorphous, necrotic and bullous vascular purpura of undetermined etiology.
